# Relationship between systemic immune inflammation index and development of complete atrioventricular block in patients with ST-elevation myocardial infarction undergoing primary percutaneous coronary intervention

**DOI:** 10.1186/s12872-024-03726-0

**Published:** 2024-01-24

**Authors:** Fatma Esin, Saban Esen, Semih Aktürk, Ömer Pekersen, Tuncay Kiris, Mustafa Karaca

**Affiliations:** 1 Atatürk Training and Research Hospital, Department of Cardiology, Izmir Katip Çelebi University, Izmir, Turkey; 2 Department of Cardiology, Tunceli State Hospital, Tunceli, Turkey

**Keywords:** Systemic immune inflammation index, Complete atrioventricular block, ST-elevation myocardial infarction, Primary percutaneous coronary intervention

## Abstract

**Background:**

The systemic immune-inflammation index (SII), based on white blood cell, neutrophil, and platelet counts, is a proposed marker of systemic inflammation and immune activation. This study aimed to explore the relationship between SII and complete atrioventricular block (CAVB) development in STEMI patients undergoing primary PCI.

**Methods:**

We retrospectively analyzed data from 883 patients who underwent primary PCI for STEMI between January 2009 and December 2017. Patients were categorized into two groups based on CAVB development. SII levels were calculated from blood samples taken on admission.

**Results:**

Of the included patients, 48 (5.03%) developed CAVB. SII was higher in patients with CAVB compared to those without CAVB (1370 [1050–1779]x109/L vs. 771 [427–1462] x109/L, *p* < 0.001). Multivariate analysis showed a significant positive correlation between SII and the risk of CAVB development (OR:1.0003, 95%CI:1.0001–1.0005, *P* = 0.044). The cut-off value for the SII in the estimation of CAVB was 1117.7 × 10^9^/L (area under the ROC curve [AUC]: 0.714, 95% CI = 0.657–0.770 with a sensitivity of 70.8% and specificity of 65.6%, *p* < 0.001).

**Conclusion:**

This study showed a significant link between high SII levels and CAVB development in STEMI patients undergoing PCI. Our findings suggest that SII may be a valuable, routinely available, and inexpensive marker for identifying patients at increased risk of CAVB.

## Introduction

Cardiovascular diseases (CVDs) continue to be the leading cause of mortality globally, with acute myocardial infarction (AMI) being a significant contributor to this burden. Among the various types of AMI, ST-elevation myocardial infarction (STEMI) is particularly severe, often requiring immediate intervention through primary percutaneous coronary intervention (PCI) as the preferred reperfusion strategy [1]. However, STEMI patients undergoing primary PCI are at risk of developing complications such as complete atrioventricular block (CAVB), which is associated with poor prognosis [2]. The overall incidence of high-grade AV block is reported to be 3–13% [2].

In recent years, the role of systemic inflammation in the pathogenesis of CVDs and AMI has gained attention [3]. Various indices have been developed to measure systemic inflammation, including the fibrinogen-to-albumin ratio and the systemic immune-inflammation index (SII) [4]. SII, calculated from peripheral lymphocyte, neutrophil, and platelet counts, has been shown to predict contrast-induced nephropathy in STEMI patients undergoing primary PCI [5]. Moreover, the effectiveness of SII in predicting the no-reflow phenomenon has been explored [6]. A recent study has shown that SII was an independent predictor of newly diagnosed reverse-dipper hypertensive patients [7]. There was a significant association between SII levels and hyperlipidemia in National Health and Nutrition Examination Survey (NHANES) adult participants [8]. It has been shown that the systemic immune inflammation index and system inflammation response index were both independently related to the presence of atrial fibrillation in patients with stroke [9].

In a previous study by Altunova et al., SII was associated with a high residual SYNTAX score in patients with ST-segment elevation myocardial infarction undergoing primary percutaneous coronary intervention [10]. Emerging evidence suggests that dynamic fluctuations in biomarkers like SII could be valuable predictors of long-term adverse cardiovascular events in STEMI patients after undergoing PCI [11]. Despite these advances, the relationship between SII and the development of CAVB in STEMI patients undergoing primary PCI remains underexplored.

This study aimed to fill this gap by investigating the relationship between SII and the development of CAVB in patients with STEMI undergoing primary PCI.

## Methods

### Study design

This study is a retrospective, single-centre cohort study conducted at the Department of Cardiology, Katip Celebi University, Ataturk Education and Research Hospital. The study was approved by the Institutional Review Board and was conducted by the principles outlined in the Declaration of Helsinki.

The study population consisted of 883 consecutive patients admitted to our institution’s cardiac catheterization laboratory with a diagnosis of ST-elevation myocardial infarction (STEMI) between January 2009 and December 2017. Patients were included if they met the following criteria: (1) diagnosed with STEMI based on clinical presentation, electrocardiographic changes, and elevated cardiac biomarkers; (2) underwent primary Percutaneous Coronary Intervention (PCI); and (3) had available pre-procedural complete blood count (CBC) data for calculation of the Systemic Immune Inflammation Index (SII). Patients with autoimmune diseases, hematologic disorders, or active infections were excluded from this study.

### Data collection

Electronic medical records were retrospectively reviewed to collect relevant clinical and laboratory data. Demographic characteristics, medical history, medication use, and procedural details were recorded. Laboratory parameters, including complete blood count, were obtained from the hospital’s laboratory database.

### Calculation of systemic immune inflammation index (SII)

Systemic Immune Inflammation Index (SII) was calculated using the formula: SII = Platelet count × (Neutrophil count / Lymphocyte count). The platelet, neutrophil, and lymphocyte counts were obtained from the pre-procedural complete blood count.

### Outcome assessment

The primary outcome of this study was the development of a CAVB, occurring either before cardiac catheterization or before hospital discharge. CAVB was defined as a complete absence of atrioventricular conduction resulting in a ventricular rate that was independent of the atrial rate, confirmed by continuous electrocardiographic monitoring and evaluated by a cardiologist. The patients were divided into two groups according to whether CAVB developed or not; CAVB (+) ( *n* = 48) or CAVB (-) (*n* = 835). Considering the CAVB rates, the study needed to recruit 771 participants to have 80% power with a 5% type 1 error level.

### Statistical analysis

Descriptive statistics were used to summarize the baseline characteristics of the study population. Continuous variables were presented as mean ± standard deviation or median with interquartile range depending on their distribution, while categorical variables were presented as frequencies and percentages. The normality of the data was assessed using the Shapiro-Wilk test. A correlation analysis between SII and other variables was conducted using the Pearson correlation test. The association between SII and the development of CAVB was assessed using logistic regression analysis, adjusting for potential confounding variables such as age, gender, and comorbidities. The predictive values of neutrophil to lymphocyte ratio (NLR), platelet, and SII were estimated by the areas under the receiver operating characteristic curve (ROC). All statistical analyses were conducted using SPSS 26 (SPSS Inc., Chicago, IL, USA). A two-sided p-value < 0.05 was considered statistically significant.

## Results

### Baseline characteristics

A total of 883 STEMI patients were included in the study. 15 patients developed CAVB before PCI or at admission, and 33 patients developed CAVB after PCI or before hospital discharge. The baseline demographic and clinical characteristics of the patients are presented in Table 1. CAVB was more common in patients who were female and older. The histories of hypertension, diabetes mellitus, hyperlipidemia, and previous coronary artery disease (CAD) were similar between groups (Table 1). Chronic kidney disease was more frequent in CAVB patients compared with those without (19% vs. 6%, *p* < 0.001).

**Table 1:** Baseline characteristics of the study population

Variables	CAVB (-) (n = 835)	CAVB (+) (n = 48)	*p*-value	
Age	59.1 ± 12.7	70.7 ± 10.7	< 0.001	
Male, n (%)	687 (82)	30 (63)	0.001	
Diabetes mellitus, n (%)	209 (25)	16 (33)	0.199	
Hypertension, n (%)	335 (40)	22 (46)	0.433	
Previous CAD, n (%)	172 (21)	9 (19)	0.758	
Previous CHF, n (%)	37 (4)	3 (6)	0.556	
Chronic kidney disease, n (%)	50 (6)	9 (19)	< 0.001	
Previous stroke, n (%)	46 (6)	3 (6)	0.827	
Hyperlipidemia, n (%)	112 (13)	6 (13)	0.857	
Multivessel disease, n (%)	419 (51)	31 (65)	0.219	
Smoking, n (%)	431 (52)	18 (38)	0.057	
Inotrope usage, n (%)	55 (7)	17 (35)	< 0.001	
Killip class > 2, n (%)	102 (12)	23 (48)	< 0.001	
IABP usage, n (%)	55 (7)	17 (3575)	< 0.001	
Thrombus aspiration device, n (%)	140 (17)	20 (42)	< 0.001	
GpIIB3A inhibitors, n (%)	293 (35)	22 (46)	0.131	
Final TIMI-3 flow n (%)	745 (89)	36 (75)	0.003	
Infarct related artery, n (%)			< 0.001	
LMCA, n (%)	9 (1)	2 (4)		
LAD, n (%)	374 (45)	9 (19)		
CX, n (%)	92 (11)	3 (6)		
RCA, n (%)	275 (33)	32 (67)		
Others n (%)	59 (7)	1 (2)		
Medical treatment at discharge, n (%)	90 (51)	19 (66)	0.159	
Aspirin n (%)	738 (97)	26 (90)	0.016	
Clopidogrel, n (%)	523 (69)	22 (73)	0.600	
Prasuqrel, n (%)	64 (8)	0 (0)	0.097	
Tigacrelor, n (%)	151 (20)	5 (17)	0.661	
Beta blockers, n (%)	670 (88)	18 (60)	< 0.001	
ACE/ARB, n (%)	627 (83)	18 (64)	0.048	
Statin n (%)	708 (93)	26 (90)	0.726	
Outcomes				
In-hospital mortality, n (%)	75 (9)	22 (46)	< 0.001	
Long-term mortality, n (%)	164 (20)	13 (27)	0.210	

**Abbreviations**: CAD: coronary artery disease, CHF: chronic heart failure, CX: Circumflex artery, LAD: Left anterior descending artery, RCA: right coronary artery, IABP: intra-aortic baloon pump; ACE-I/ARB: angiotensin-converting enzyme inhibitors/ angiotensin receptor blocker, TIMI: Thrombolysis in myocardial infarction

The culprit lesion was the right coronary artery in most CAVB patients (67% vs. 33%, *p* < 0.001). Inotrope and intra-aortic balloon pump usage were more common in CAVB patients than in those without (Table 1). CAVB patients had a higher Killip class than those without CAVB (48% vs. 12%, *p* < 0.001).

The laboratory findings are presented in Table 2. WBC and neutrophil counts were higher in CAVB patients (13.1 ± 4.2 vs. 11.8 ± 3.8, *p* = 0.019; 10.1 ± 3.7 vs. 8.1 ± 3.6, *p* < 0.001, respectively). Lymphocyte count was lower in patients with CAVB compared to those without (2.1 ± 0.8 vs. 2.7 ± 1.6, *p* < 0.001). The patients with CAVB had lower LVEF than patients without CAVB (42.8 ± 9.2% vs. 46.3 ± 10.0%, *p* = 0.021). SII was higher in CAVB patients (1370 [1050–1779]x10^9^/L vs. 771 [427–1462] x10^9^/L, *p* < 0.001, Table 2). The correlations of SII with other variables were presented in Table 3.

**Table 2:** Laboratory findings of the patients before matching

Variables	CAVB (-) (*n* = 835)	CAVB (+) (*n* = 48)	***p***-value
WBC count (×10^3^/µL)	11.8 ± 3.8	13.1 ± 4.2	0.019
Hemoglobin (g/dl)	14.1 ± 1.9	12.4 ± 1.9	< 0.001
eGFR* (mL/min/1.73m^2^)	90 (73–108)	67 (37–82)	< 0.001
Platelet count (×10^3^/µL)	267 ± 77	313 ± 110	< 0.001
Neutrophil (×10^3^/µL)	8.1 ± 3.6	10.1 ± 3.7	< 0.001
Lymphocyte (×10^3^/µL)	2.7 ± 1.6	2.1 ± 0.8	< 0.001
Total cholesterol (mg/dL)	184 ± 48	166 ± 46	0.050
LDL-C (mg/dL)	114.5 ± 37.9	102.8 ± 36.3	0.110
HDL-C (mg/dL)	37.1 ± 11.7	37.5 ± 15.9	0.852
Trigliseride * (mg/dL)	141 (102–195)	115 (92–182)	0.168
LVEF *	46.3 ± 10.0	42.8 ± 9.2	0.021
SII* (x10^9^/L)	771 (427–1462)	1370 (1050–1779)	< 0.001

**Abbreviations**: WBC; white blood cell, eGFR: estimated glomerular filtration rate, LVEF: left ventricular ejection fraction, SII; systemic immune-inflammatory index, LDL-C: low-density lipoprotein cholesterol, HDL-C: high-density lipoprotein cholesterol

* Comparison was made using the Mann-Whitney *U* test at *p* < 0.05, and these values were described by a median with an interquartile range (25th and 75th percentile)

**Table 3:** The correlations of SII with other variables

Variables	r	***p***-value
Age (years)	0.172	< 0.001
LVEF (%)	-0.175	< 0.001
Killip class	0.189	< 0.001
Final TIMI flow	-0.104	0.002
Total cholesterol (mg/dl)	-0.103	0.004
LDL-C (mg/dl)	-0.078	0.034
HDL-C (mg/dl)	0.011	0.754
Trigliseride (mg/dl)	-0.100	0.006
Haemoglobin (mg/dl)	-0.270	< 0.001
eGFR	-0.124	< 0.001

**Abbreviations**: LDL: low-density lipoprotein cholesterol; HDL-C: HDL-C;high-density lipoprotein cholesterol, eGFR: estimated glomerular filtration rate, LVEF: left ventricular ejection fraction, TIMI: Thrombolysis in myocardial infarction

### Association between SII and CAVB

**Fig. 1:**
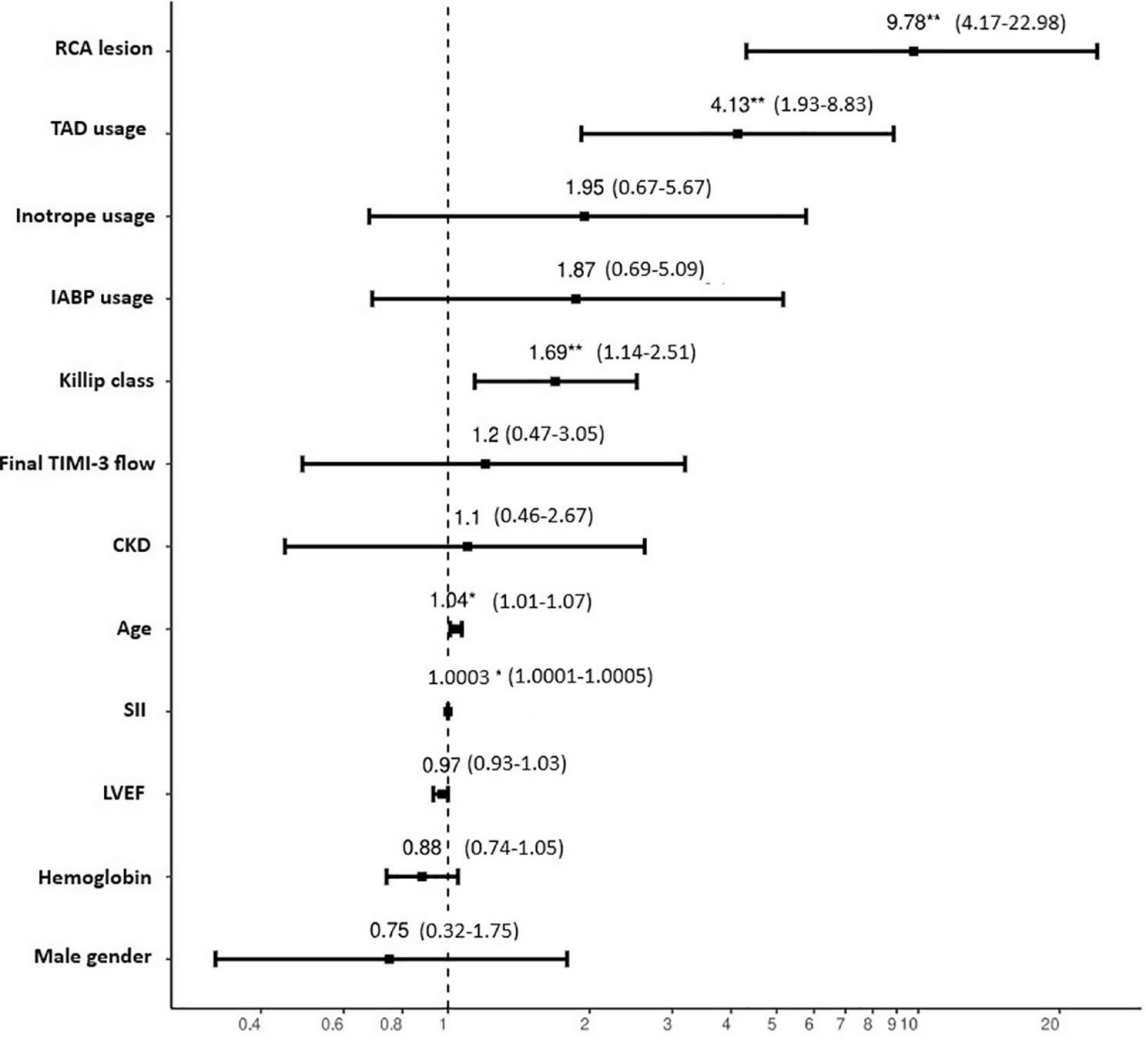
The predictors of the development of complete atrioventricular block (CAVB)

Logistic regression analysis was used to assess the association between SII and the development of CAVB, adjusting for potential confounding variables such as age, sex, comorbidities, and procedural characteristics. After adjusting for these variables, SII was found to be an independent predictor of the risk of developing CAVB (OR:1.0003, 95%CI:1.0001–1.0005, *P* = 0.044, Fig. 1).

**Fig. 2:**
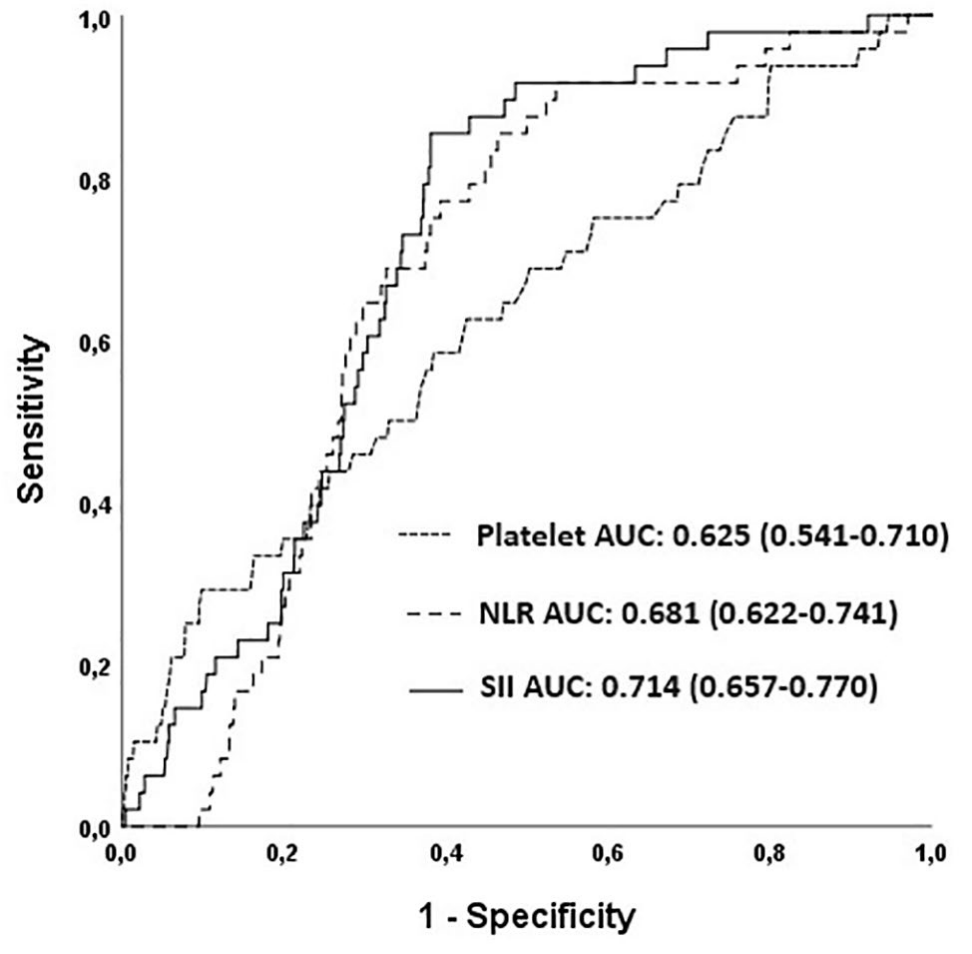
Receiver operating characteristic (ROC) curves for the neutrophil-to-lymphocyte ratio (NLR), platelet, and systemic immune-inflammation index (SII) for predicting complete atrioventricular block (CAVB)

ROC analysis (Fig. 2) revealed that SII > 1117.7 × 10^9^/L was predictive with a sensitivity of 70.8% and specificity of 65.6% (area under the ROC curve [AUC]: 0.714, 95% CI = 0.657–0.770, *p* < 0.001, Fig. 2) for predicting CAVB. The value of SII in predicting CAVB was better than neutrophil to lymphocyte ratio (NLR) alone (AUC:0.714 vs. 0.681, z = 2.001, p-value for the difference = 0.045) and PLT alone (AUC:0.714 vs. 0.625, z = 2.290, p-value for the difference = 0.022, Fig. 2).

The patients with CAVB had a higher in-hospital mortality rate (46% vs. 9%, *p* < 0.001). However, the long-term mortality rate was not different between groups (27.1% vs. 19.6%, *p* = 0.210).

## Discussion

To the best of our knowledge, this is the first study to investigate the association of SII with CAVB in STEMI patients who underwent PCI. Our findings showed a significant association between higher SII levels and an increased risk of CAVB development in this patient population.

The SII is a composite index calculated using the counts of peripheral lymphocytes, neutrophils, and platelets. It is considered a novel marker for inflammation and immune response. In other words, it is a numerical representation of certain components of the immune and inflammatory systems in the body [12]. There may be a connection between some factors represented by SII and the development of CAVB. This connection could involve the influence of gap junctions and the immune-inflammatory system on atrioventricular conduction [13–16]. However, the underlying mechanism linking SII and CAVB development remains unclear. Inflammatory cells and IL-6, a pro-inflammatory cytokine, can damage the conduction system, culminating in the development of CAVB [17, 18]. This suggests that the association between SII and CAVB development may be mediated, at least in part, by IL-6. IL-6 can affect the electrical activity of cardiac muscle cells by altering the function of ion channels, which can lead to abnormalities in cardiac conduction and ultimately contribute to the development of CAVB. A recent study showed that IL-6 potently inhibited connexins (specifically Cx40 and Cx43), which are proteins that form gap junctions, facilitating communication between adjacent cells, in cultured cardiomyocytes and macrophages [19, 20]. The inhibitory effect of IL-6 on connexins was reversed when cells were preincubated with a monoclonal anti-IL-6 antibody [19]. This suggests that the antibody could neutralise or block the effects of IL-6 on connexins. In guinea pigs, the injection of IL-6 was associated with a slowing of AV conduction, as indicated by the prolongation of the PR interval and PR-segment [20, 21]. Additionally, there was an increased susceptibility to drug-associated severe bradyarrhythmia, including complete AV dissociation and asystole [21]. Therefore, there have been recent studies confirming the important role of excess IL-6 in the development of CAVB, and there is a suggestion that IL-6 antagonists like Anakinra could be a potential therapeutic option. However, it is important to consider the potential roles of other inflammatory cytokines, oxidative stress, and autoimmune responses. Tumour necrosis factor alpha (TNF-α) and CRP as inflammatory markers have also been shown to be associated with CAVB development [19, 22]. Therefore, the relationship between SII and CAVB development may also be regulated by inflammatory mediators other than IL-6.

Oxidative stress, which results from an imbalance between the production of reactive oxygen species (ROS) and the body’s antioxidant defence system, has been implicated in the pathogenesis of various cardiovascular diseases, including atherosclerosis, and hypertension [23, 24]. Also, it has been shown that oxidative stress and autoimmune responses may also play a role in the development of CAVB. A study by Lazzerini et al. found that patients with rheumatoid arthritis, a systemic inflammatory disease, had a higher risk of developing arrhythmic risk compared to the general population [25].

It is important to consider the potential impact of age on the development of CAVB. In previous studies, age was found to be an independent predictor of CAVB in STEMI patients [26, 27]. Similar to the results of these studies, age was independently associated with the development of AV block in these patients in our study. It has been shown that the responsible lesion in most patients with AV block was RCA [26, 27]. In our study, the culprit lesion was found to be RCA in these patients. Also, it was independently related to the development of CAVB. The Killip class was found to be a predictor of CAVB in acute coronary syndrome patients in a study published by Santos et al. [28]. We showed that a higher Killip class was associated with a higher risk of CAVB in STEMI patients treated with PCI. Although female gender was an independent predictor of the development of CAVB in previous studies [27, 28], it was not significant in multivariate analysis in our study.

The presence of CAVB was associated with in-hospital mortality in the acute coronary syndrome setting [29]. Different results have been reported in the related literature regarding the impact of CAVB on long-term mortality in the setting of ACS [26–28]. Kawamura et al. showed that CAVB in nonanterior STEMI was associated with long-term mortality [29]. In another study, it was not an independent predictor of mortality in ACS patients [28]. CAVB patients had a higher rate of in-hospital mortality, however long-term mortality rate was not different between groups in the presented study.

It is worth noting that our study has several limitations. It is a single-centre, retrospective study with a relatively small sample size, which may limit the generalizability of our findings. The retrospective nature of our study may have introduced selection bias, and the small sample size may have limited our ability to detect smaller effect sizes. We did not assess other inflammatory markers, such as TNF-α and IL-6, which may also be associated with CAVB development. Additionally, we cannot rule out the presence of unmeasured confounders, which could have influenced the development of CAVB. The results of this study should be considered as hypothesis generating. Therefore, future research may address these issues to gain a more comprehensive understanding of the relationship between SII and the development of CAVB in these patients.

## Conclusion

Our study suggested that a higher SII level was associated with an increased risk of developing CAVB in patients with STEMI undergoing primary PCI. In cases where atrioventricular block is exacerbated or triggered by systemic inflammation, it might be beneficial to promptly and specifically manage the inflammatory process. This implies that controlling inflammation could potentially alleviate or improve the conduction disturbance seen in severe atrioventricular blocks. This could involve using anti-inflammatory medications or treating the underlying cause of systemic inflammation.

## Data Availability

The data supporting this study’s findings are available from the corresponding author upon reasonable request.

## References

[1] Ibanez B, James S, Agewall S, Antunes MJ, Bucciarelli-Ducci C, Bueno H (2018). 2017 ESC guidelines for the management of acute myocardial infarction in patients presenting with ST-segment elevation: the Task Force for the management of acute myocardial infarction in patients presenting with ST-segment elevation of the European Society of Cardiology (ESC). Eur Heart J.

[2] Harpaz D, Behar S, Gottlieb S, Boyko V, Kishon Y, Eldar M (1999). Complete atrioventricular block complicating acute myocardial infarction in the thrombolytic era. SPRINT Study Group and the Israeli Thrombolytic Survey Group. Secondary Prevention Reinfarction Israeli Nifedipine Trial. J Am Coll Cardiol.

[3] Libby P, Ridker PM, Maseri A (2002). Inflammation and atherosclerosis. Circulation.

[4] Liu G, Fan CM, Guo H, Fan WN, Li ML, Cui GX (2021). Fibrinogen-to-albumin ratio predicts long-term outcomes for patients with ST-elevation myocardial infarction and multivessel disease: a prospective observational cohort study. Exp Ther Med.

[5] Karauzum I, Karauzum K, Hanci K, Gokcek D, Kalas B, Ural E (2022). The utility of systemic Immune-inflammation index for Predicting contrast-Induced Nephropathy in patients with ST-Segment Elevation myocardial infarction undergoing primary percutaneous coronary intervention. Cardiorenal Med.

[6] Ozturk E, Esenboga K, Kurtul A, Kilickap M, Karaagaoglu E, Karakaya J (2023). Measurement of uncertainty in Prediction of No-Reflow Phenomenon after primary percutaneous coronary intervention using systemic Immune inflammation index: the Gray Zone Approach. Diagnostics (Basel).

[7] Karakayali M, Omar T, Artac I, Rencuzogullari İ, Karabag Y, Demir O (2023). The relationship between the systemic immune-inflammation index and reverse-dipper circadian pattern in newly diagnosed hypertensive patients. J Clin Hypertens (Greenwich).

[8] Mahemuti N, Jing X, Zhang N, Liu C, Li C, Cui Z, Liu Y, Chen J (2023). Association between systemic immunity-inflammation index and hyperlipidemia: a Population-based study from the NHANES (2015–2020). Nutrients.

[9] Lin K, Cai FF. Mq. The systemic immune inflammation index and system inflammation response index are potential biomarkers of atrial fibrillation among the patients presenting with ischemic stroke. Eur J Med Res 2022 27:106. 10.1186/s40001-022-00733-9 PMC9250264 35780134

[10] Altunova M, Karakayalı M, Kahraman S, Avcı Y, Demirci G, Sevinç S, Yazan S, Şahin A, Ertürk M (2023). Systemic Immune-Inflammatory Index is Associated with residual SYNTAX score in patients with ST-Segment Elevation myocardial infarction. Anatol J Cardiol.

[11] Liu Y, Liu J, Liu L, Cao S, Jin T, Chen L (2023). Association of systemic inflammatory response index and pan-immune-inflammation-value with long-term adverse Cardiovascular events in ST-Segment Elevation myocardial infarction patients after primary percutaneous coronary intervention. J Inflamm Res.

[12] Hu B, Yang XR, Xu Y, Sun YF, Sun C, Guo W (2014). The systemic immune-inflammation index predicts prognosis of patients after curative resection for hepatocellular carcinoma. Clin Cancer Res.

[13] Delmar M, Makita N (2012). Cardiac connexins, mutations and arrhythmias. Curr Opin Cardiol.

[14] Ellinor PT, Jameson HS (2017). Connexin-45 as a New Gene underlying syndromic AV block. J Am Coll Cardiol.

[15] Rosenthal N (2017). A Guardian of the heartbeat. N Engl J Med.

[16] Swirski FK, Nahrendorf M (2018). Cardioimmunology: the immune system in cardiac homeostasis and disease. Nat Rev Immunol.

[17] Dobrzynski H, Anderson RH, Atkinson A, Borbas Z, D’Souza A, Fraser JF (2013). Structure, function and clinical relevance of the cardiac conduction system, including the atrioventricular ring and outflow tract tissues. Pharmacol Ther.

[18] Hulsmans M, Clauss S, Xiao L, Aguirre AD, King KR, Hanley A (2017). Macrophages Facilitate Electrical Conduction in the Heart Cell.

[19] Lazzerini PE, Laghi-Pasini F, Acampa M, Srivastava U, Bertolozzi I, Giabbani B (2019). Systemic inflammation rapidly induces reversible Atrial Electrical remodeling: the role of Interleukin-6-Mediated changes in Connexin expression. J Am Heart Assoc.

[20] Lazzerini PE, Acampa M, Cupelli M, Gamberucci A, Srivastava U, Nanni C, Bertolozzi I, Vanni F, Frosali A, Cantore A, Cartocci A, D’Errico A, Salvini V, Accioli R, Verrengia D, Salvadori F, Dokollari A, Maccherini M, El-Sherif N, Laghi-Pasini F, Capecchi PL, Boutjdir M (2021). Unravelling atrioventricular Block Risk in Inflammatory diseases: systemic inflammation acutely delays atrioventricular conduction via a cytokine-mediated inhibition of Connexin43 expression. J Am Heart Assoc.

[21] Zhu X, Wang Y, Xiao Y (2022). Arrhythmogenic mechanisms of interleukin-6 combination with hydroxychloroquine and azithromycin in inflammatory diseases. Sci Rep.

[22] Fernandez-Cobo M, Gingalewski C, Drujan D, De Maio A (1999). Downregulation of connexin 43 gene expression in rat heart during inflammation. The role of tumour necrosis factor. Cytokine.

[23] Kaya MG, Yarlioglues M, Gunebakmaz O, Gunturk E, Inanc T, Dogan A (2010). Platelet activation and inflammatory response in patients with non-dipper hypertension. Atherosclerosis.

[24] Lazzerini PE, Capecchi PL, Laghi-Pasini F (2017). Systemic inflammation and arrhythmic risk: lessons from rheumatoid arthritis. Eur Heart J.

[25] Heistad DD, Arteriosclerosis, Thrombosis, Biology V. 2005. Arteriosclerosis, Thrombosis, and Vascular Biology. 2005;25(1):2–4.

[26] Kosmidou I, Redfors B, Dordi R (2017). Incidence, predictors, and outcomes of high-Grade Atrioventricular Block in patients with ST-Segment Elevation myocardial infarction undergoing primary percutaneous coronary intervention (from the HORIZONS-AMI Trial). Am J Cardiol.

[27] Gang UJ, Hvelplund A, Pedersen S (2012). High-degree atrioventricular block complicating ST-segment elevation myocardial infarction in the era of primary percutaneous coronary intervention. Europace.

[28] Santos H, Santos M, Almeida I (2021). Portuguese Registry of Acute Coronary syndromes. High-grade atrioventricular block in acute coronary syndrome: Portuguese experience. J Electrocardiol.

[29] Kawamura Y, Yokoyama H, Kitayama K (2021). Clinical impact of complete atrioventricular block in patients with ST-segment elevation myocardial infarction. Clin Cardiol.

